# Results of an international survey on the status of prehospital care

**DOI:** 10.1177/17474930231177204

**Published:** 2023-05-30

**Authors:** Anthony G Rudd, Jing Zhao, Gary Ford, Rita Melifonwu, Siju V Abraham, Marc Fisher, Grethe Andersen, David Waters, Dou Li, Renyu Liu

**Affiliations:** 1 Stroke Research Group and Division for Health & Social Care Research, Kings College London, London, UK; 2 Coalition of Stroke Taskforces for Stroke; 3 Department of Neurology, Minhang Hospital, Fudan University, Shanghai, China; 4 Radcliffe Department of Medicine, University of Oxford and Oxford University Hospitals NHS Foundation Trust, Oxford, UK; 5 Department of Nursing Science, Nnamdi Azikiwe University, Awka, Nigeria; 6 Life after Stroke Centre, Stroke Action Nigeria, Onitsha, Nigeria; 7 Department of Emergency Medicine, Jubilee Mission Medical College and Research Institute, Thrissur, India; 8 Department of Neurology, Beth Israel Deaconess Medical Center, Harvard Medical School, Boston, MA, USA; 9 Departments Clinical Medicine and Neurology, Aarhus University, Aarhus, Denmark; 10 Council of Ambulance Authorities, Hilton, SA, Australia; 11 Department of Emergency Medicine, Beijing Emergency Medical Center, Beijing, China; 12 Departments of Anaesthesiology and Critical Care and Neurology, Perelman School of Medicine, University of Pennsylvania, Philadelphia, PA, USA; 13 World Stroke Organisation Taskforce on Prehospital Care, Geneva Switzerland

**Keywords:** Stroke, emergency medical services, prehospital, training, quality of care, international

## Abstract

**Background:**

Prehospital care including recognition of stroke symptoms by the public and professionals combined with an efficient and effective emergency medical service (EMS) is essential to increase access to effective acute stroke care. We undertook a survey to document the status of stroke prehospital care globally.

**Methods:**

A survey was distributed via email to the World Stroke Organization (WSO) members. Information was sought on the current status of stroke prehospital delay globally, including (1) ambulance availability and whether payment for use is required, (2) ambulance response times and the proportion of patients arriving at hospital by ambulance, (3) the proportion of patients arriving within 3 h and more than 24 h after symptom, (4) whether stroke care training of paramedics, call handlers, and primary care staff, (5) availability of specialist centers, and (6) the proportion of patients taken to specialist centers. Respondents were also asked to identify the top three changes in prehospital care that would benefit their population. Data were analyzed descriptively at both country and continent level.

**Results:**

Responses were received from 116 individuals in 43 countries, with a response rate of 4.7%. Most respondents (90%) reported access to ambulances, but 40% of respondents reported payment was required by the patient. Where an ambulance service was available (105 respondents) 37% of respondents reported that less than 50% of patients used an ambulance and 12% less than 20% of patients used an ambulance. Large variations in ambulance response times were reported both within and between countries. Most of the participating high-income countries (HIC) offered a service used by patients, but this was rarely the case for the low- and middle-income countries (LMIC). Time to admission was often much longer in LMIC, and there was less access to stroke training for EMS and primary care staff.

**Conclusions:**

Significant deficiencies in stroke prehospital care exist globally especially in LMIC. In all countries, there are opportunities to improve the quality of the service in ways that would likely result in improved outcomes after acute stroke.

## Introduction

Accessing acute stroke services that deliver reperfusion therapies and early stroke unit care requires effective recognition of stroke symptoms and prehospital ambulance services that deliver patients rapidly to hospitals able to deliver stroke care.

Compared with research on the management of acute stroke in hospital, there has been much less attention paid to identifying ways to improve prehospital care. This is despite the fact that even in well-developed health systems in high-income countries (HIC), it is still unusual for more than 50% of patients to arrive at a hospital capable of delivering revascularization treatments within 3 h of the onset of the symptoms.^1,2^ Only a few studies have addressed ways to improve the recognition of stroke symptoms by first responders and improved communication between paramedics and stroke specialists.^3–8^ The evidence base is now fairly strong for mobile stroke units (MSUs), but these are resource intensive to establish and are dependent on a strong hospital stroke service for support.^9^ They have not been tested thoroughly in rural areas and are probably of little relevance in low- and middle-income countries (LMIC). A few trials have been conducted on delivering acute therapy such as blood pressure management in the ambulance,^10,11^ but so far nothing has been shown to change outcomes. Reorganization of regional stroke services, including centralization of specialist care supported by paramedics, is key to ensuring that patients are taken to the correct hospital, such as the model developed in London which has been shown to improve outcomes and save resources.^12,13^ However, the most important elements for the patient are recognizing the stroke and that treatment is possible if responded to urgently with maintenance of homeostasis, including oxygenation and blood glucose and transporting the patient as quickly as possible to a place where the diagnosis can be confirmed, and definitive treatment delivered.

It is known that the quality and quantity of prehospital care for all acutely ill patients varies enormously around the world, ranging from no prehospital emergency care to sophisticated well organized and trained paramedic services.^14,15^ There is little evidence that there has been much progress since the World Health Organization passed Resolution 60.22 “Health Systems: Emergency Care Systems,” in 2011.^16^

The aim of this study was to document the status and establish the key issues in the delivery of prehospital care for patients with acute stroke and to gain the insight of local clinicians as to the key prehospital issues that need to be resolved to improve the care of their patients. The World Stroke Organization established a Taskforce on Prehospital Care in 2022 to work on improving care in the countries represented in the organization. To inform its program of work, it was necessary to establish the key issues, and here we report results of a survey conducted in 43 countries.

## Methods

A brief questionnaire was developed by the members of the World Stroke Organization Taskforce on Prehospital Care (WSOTPC) with the areas covered summarized in Table 1. The questionnaire was written in English and kept very short to maximize the response rate. The questions were put into a Survey Monkey electronic format and circulated by the WSO Administrative office to WSO members in April 2022 with reminders sent in May and June 2022. We phrased the questions carefully to reduce the chances of possible misinterpretation, particularly by people whose first language is not English. It was piloted among the members of the WSPTPC. It was accepted at the outset that the survey would only achieve a relatively small sample of the worldwide picture but given the paucity of data currently available it was still considered to be valuable.

**Table 1.** The questions included in the survey.

Is there an ambulance service, and if so what proportion of patients with stroke use it? Does use of the ambulance require payment by the patient? What is the average response time of the ambulance services to suspected stroke patient (alert to scene time), in the last 1 year? Are there specialist centers for the treatment of stroke, and if so what proportion of patients are taken directly to them? What proportion of patients arrive within 3 h of the onset of symptoms and what proportion arrive after 24 h? What if any training is available on stroke to emergency call handlers, paramedics, and primary care clinicians? What three changes would make the biggest difference in improving prehospital care for their patients.

All 2452 World Stroke Organization members were emailed with a link to the survey and asked to respond on behalf of their local area, region, or nation. Two further reminders were sent at approximately monthly intervals. The responses were taken at face value as there was no way of systematically validating the answers.

Responding to the survey was voluntary and no individual patient information was requested, so ethical approval for the study was not required.

Data were returned by the WSO Administrative Office as an Excel spreadsheet. Descriptive analysis of the data was performed and reported at a national and continent level. The free text question asking for the three most important changes that the respondent thought would be most important to improve their service was analyzed by identifying the range of topics being suggested and then allocating each of the responses into one of these topics. The 10 most popular responses are reported.

High-, middle-, and low-income countries were classified using World Bank definitions.^17^

## Results

**Figure 1.**
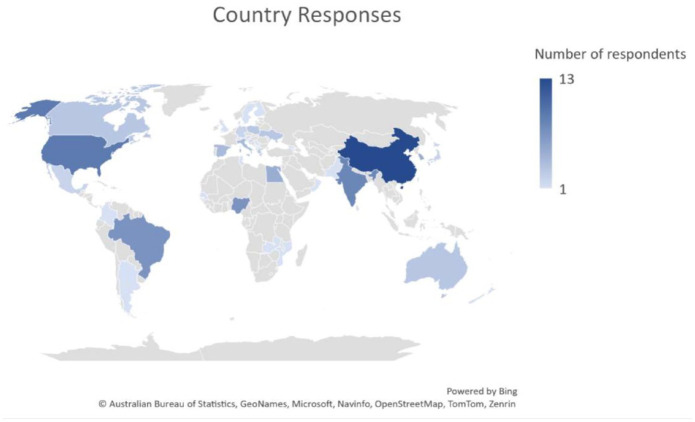
World map showing participating countries and region, and the frequency of responses per country or region.

**Figures 2.**
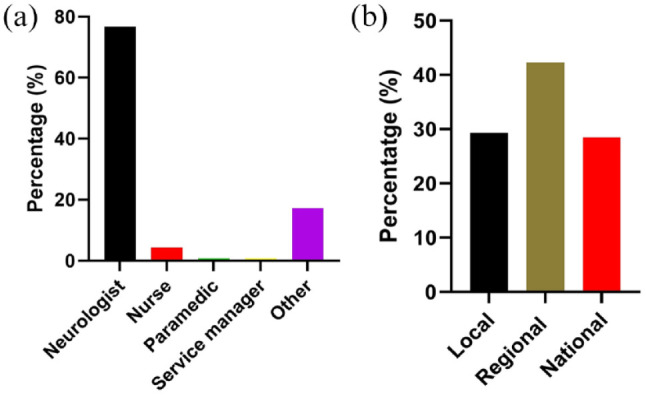
(a) The composition of the survey responders. (b) Proportions responding at local, regional, or national level.

A total of 116 responses out of 2452 people emailed (4.7%) were collected from 43 countries, which accounted for 22% of the world’s 196 countries. Of these, 22 out of 67 responses (32.8%) were from HIC and 21 out of 128 responses (16.4%) were from LMIC (Figure 1). Among the responders, 88 (77%) were neurologists, as shown in Figure 2(a). Thirty-three (28.5%) of the responders provided comments on national issues, 49 (42.2%) provided comments on regional issues, and 34 (29.3%) reported local data, as depicted in Figure 2(b). Supplementary Table 3 provides responses to the questions by country, with missing data labeled as “Not known.” If there were multiple responses per country and data were missing, the figure presented was based on available data from at least one respondent.

## Ambulance provision

One hundred five respondents (90.5%) reported that stroke patients have access to ambulances. However, 47 of these respondents (40.5%) noted that patients are required to pay for ambulance services. Out of 105 respondents who have ambulance services, 39 (37%) reported that less than half of stroke patients arrived at the hospital via ambulance. In 13 (12%) of these cases, less than 20% of patients used ambulance services. The response times from alert to arrival on the scene varied significantly between and within countries, as presented in Supplementary Table 3. Thirty-four out of 43 (79%) countries reported a maximum ambulance response time of less than 60 min, while 22 (51%) countries had a response time of 30 min or less. The study also found that there was a significant difference between LMIC and HIC in the reported access and use of ambulances. Among the 23 returns from African countries, 14 (63%) reported that less than 20% of patients arrived by ambulance, while 4 (18%) reported that greater than 20% arrived by ambulance (4 not known). Conversely, in North America (the United States and Canada), all 11 respondents reported over 40% of patients arrived by ambulance, with most respondents reporting rates around 80%. Of patients arriving by ambulance, out of the 35 European responses, only two reported levels of ambulance usage below 20%, two had no data, and the rest reported between 40% and 95%.

## Time from stroke to arrival at hospital

Forty (34%) respondents stated that less than 20% of patients arrived under 3 h after the onset of symptoms with 26 (22%) reporting that over 50% of patients arrived more than 24 h after the onset of the stroke. Again, a notable contrast was observed between countries with low and high income. Out of the 16 African participants who provided data for the item, 8 (50%) reported that more than 50% of patients arrived after 24 h, while in Europe, only four out of 35 (11%) responses indicated the same.

## Training of prehospital clinical staff

**Figure 3.**
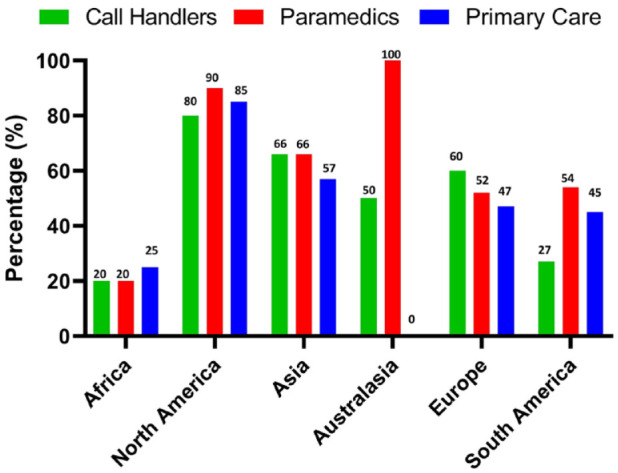
Percentage of people who had relevant training.

The results show that with few exceptions there are major gaps in the provision of training (Figure 3). Results reported from African nations noted that only 20% of areas offered training to call handlers and paramedics and 25% of areas offered training to primary care staff. In Europe the figures were 60%, 52%, and 47%, respectively. In Asian countries, 66% of areas provided training for both call handlers and paramedics and 51% for primary care staff, with the corresponding percentages for North America being 80%, 90%, and 85%; for South America 27%, 54%, and 45%; and for Australasia 50%, 100%, and 0%, respectively. Overall, if anyone is given training it is generally the paramedics rather than the call handlers. Training of primary care clinicians, although not usually included as part of the emergency medical service (EMS) is essential, given that for many patients the primary care physician or nurse will be the first point of contact when they develop symptoms.

## Continental summaries

**1. Europe:** Responses from 19 countries (Armenia, Austria, Croatia, Denmark, Finland, Georgia, Germany, Greece, Italy, Moldova, Monaco, Poland, Portugal, Serbia, Spain, Sweden, Switzerland, Ukraine, the United Kingdom) suggest that ambulance and paramedic services are generally accessible, but there is considerable variation. Stroke training for call handlers, paramedics, and primary care clinicians is clearly lacking. Some countries provide high-quality care, such as Austria, where the ambulance service is free and the average response time is 15 min, with 71% of patients arriving within 3 h of symptom onset and more than 80% being taken directly to a specialist stroke center. In the United Kingdom and Spain, ambulance services are also free with response times of around 20 min, and more than 50% of patients arrive within 3 of symptom onset. However, Greece has lower ambulance usage rates (20–35%) and longer response times (30–60 min), with fewer patients arriving within 3 h of symptom onset.
**2. South America:** The survey received responses from three countries (Argentina, Brazil, Colombia) revealing a lack of adequate ambulance services and training for emergency medical staff. In Argentina, 50% of patients arrived at hospitals by ambulance, with 50% arriving in less than 3 h and 10% after 24 h. In Brazil, 40–70% of patients used ambulance services, with response times ranging from 30 min to 6 h, and 0–60% arrived in less than 3 h, and between 10% and 70% after 24 h. In Colombia, the ambulance service requires direct payment with a response time of 1 h, and only 30% of patients arrive in less than 3 h.
**3. Australasia:** questionnaires were returned from both Australia and New Zealand. Overall, there are well organized services for prehospital care but there are significant issues in delivering care to rural areas with sparse populations.
**4. Asia:** Like other continents, Asia has significant variations between rich and poor countries in terms of stroke care. In LMIC, apart from China, there is generally a lack of ambulance services, stroke training, and specialist stroke services.
  a. Mainland China: In mainland China, from the 12 responses received, ambulance usage varies from 20% to 50%, with some areas requiring payment. Response times average around 30 min, with up to 50% of patients presenting within 3 h, and up to 70% arriving after 24 h. Most regions have specialist stroke centers. In general, training is provided to prehospital clinical staff.
  b. In LMIC Asia (India, Pakistan, Sri Lanka, Indonesia), ambulance services are often not used, resulting in late patient presentations, with up to 70% of patients arriving after 24 h.
  c. In HIC Asia (Japan, South Korea, Oman), Japan stands out for having well-organized services with up to 90% of patients arriving within 3 h of symptom onset, and less than 5% arriving after 24 h. Oman and South Korea reported to have effective services that match some of the best in North America and Europe, (although not as effective as Japan).
**5. North America (Canada and the United States):** Services are reported to be generally well developed and effective, but particularly in the United States there are issues with rural populations not being served as well as those living in cities. In some areas, 30–40% of patients are presenting more than 24 h after the onset of symptoms. Throughout North America, training is reported to be given to primary care staff as well as call handlers and paramedics.
6. In **Africa**, including Egypt, Mozambique, Nigeria, Rwanda, Senegal, Tanzania (including Zanzibar), Tunisia, and Zambia, there was very little prehospital care and specialist stroke care reported. Less than 5% of stroke patients with symptoms were transported by ambulance in Zambia, Nigeria, Mozambique, Tanzania, and Rwanda, and many patients presented late with no access to acute treatments. Tunisia and Egypt had better services, but rates reported were still low with only about 20% arriving within 3 h. There was little training provided to the prehospital clinical staff.

## Key areas for development

The issues identified for development by the survey respondents varied greatly between countries that already had reasonably well-developed services and those that had little or no services. The top 10 items identified are given in Table 2. These include better education and training for the public and clinical staff, improved communication between EMS staff and neurologists/stroke specialists, better defined pathways and more stroke centers, better funded ambulance services not requiring the direct payment by the patient, more MSUs, central government taking responsibility for developing services, measurement of quality, and more research to find ways to identify large vessel occlusion in the prehospital setting.

**Table 2.** Top 10 changes needed to improve prehospital stroke services.

1. Better quality and regular public education campaigns regarding the symptoms and signs of stroke and the importance of urgent medical care (36 suggestions) 2. Better education for EMS staff—call handlers, paramedics, and primary care. Development of smart phone apps to enable this to happen and to have telemedicine facilities in ambulances (32 suggestions) 3. Improved communication between EMS staff and neurologists/stroke specialists (31 suggestions) 4. More stroke centers and clearly defined pathways (19 suggestions) 5. A properly funded ambulance service (17 suggestions) 6. Development of more mobile stroke units (6 suggestions) 7. Removal of the need for patients to have to fund EMS before they can access the services (7 suggestions) 8. Central government action to improve emergency stroke care with national pathways, ensuring access to specialist centers and a clear stroke pathway (5 suggestions) 9. Research to develop better tools for use in prehospital to identify large vessel occlusion and enable triage of such patients to centers able to deliver endovascular treatment (4 suggestions) 10. Systems for monitoring the quality of EMS care (4 suggestions)

EMS: emergency medical services.

Others suggestions included solving traffic problems, national guidelines, educating hospital staff, better systems for inter-hospital transfer for thrombectomy, improved systems for recognizing atypical stroke, air services for difficult to reach patients, and certification of stroke centers.

## Discussion

The results of this survey show that overall, there are aspects of prehospital stroke care that should be improved ranging from more responsive and efficient ambulance services to better trained prehospital clinical staff. Services are generally better in HICs compared with LMICs, although even in HICs there are nearly always some aspects of the service that would benefit from reform.

An effective stroke service requires an educated population able to recognize when someone might be having a stroke. There should be an easy way to contact EMSs that are well staffed with trained call handlers and paramedics; sufficient ambulances and paramedics to reach patients within a few minutes; and a network of specialist hospitals that are known to the paramedics and are ready to receive and treat patients 24 h a day, 7 days a week.^18^ Primary care clinicians such as general practitioners and community nurses are often the first point of professional contact for stroke patients, and it is therefore important that they are trained in stroke recognition and immediate management. Failure to recognize the symptoms of stroke and failing to respond with urgency is undoubtedly a critical factor in delayed arrival at hospital and consequently lower reperfusion rates and increased long-term disability.^19^ It has been previously reported that novel strategies with a coordinated effort to improve stroke awareness can reduce prehospital delay significantly.^20,21^

This survey confirms that there are many countries in the world, including a large number where stroke is the most frequent cause of death and disability, that have little or no EMS available to respond to acute stroke. While these are largely in LMICs, there are also many HICs that fail to meet some or all of the criteria described above and are therefore likely to be compromising the management and outcomes of their patients.

Simple measures such as the use of standardized protocols to enable call handlers to recognize possible stroke, prioritization of stroke by paramedics, clear pathways, and stroke maps to ensure that patients are taken to the appropriate hospital and monitoring the quality of care would reduce the time from the onset of symptoms to arrival in hospital and would be likely to have a major effect at both an individual and population level in the reduction of death and disability following stroke.

One area needing development, is for greater involvement of stroke services in supporting EMS both in the organization of their stroke services and in training of staff. Improved training of prehospital clinical staff might result in improvement in the recognition of stroke by call handlers. One study in the United Kingdom showed that only 51% of stroke calls are identified as stroke at the point of dispatch.^22^ In another region of the United Kingdom, correct recognition of stroke by call handlers improved from 63% to 80% after completion of a 2-h online training package.^23^ A study from Denmark showed that improved communication between paramedics and stroke specialists using a prehospital stroke score and teleconferencing improved the quality of diagnosis particularly in identifying those patients with large vessel occlusion suitable for intra-arterial clot retrieval.^24^ The success of this strategy has led to the establishment of formal collaboration between the prehospital and the hospital services. Using audit data from Sweden, it was shown that recognition of stroke by the EMSs resulted in lower mortality.^25^

Measurement of care quality is now almost routine in the hospital,^26^ especially in HIC, but there are few services that include measurement of the prehospital quality. As a result, this critical phase of the illness remains largely hidden.

While there have been reports from individual countries and a few at a continental level on the quality of prehospital stroke care there have been few that have tried to understand the status of prehospital stroke care globally. The European Stroke Organisation conducted a survey on regional and national stroke care plans that included some aspects of prehospital care.^27^ Of the 44 countries, 37 had some sort of care plan in place with 79% of these having formal links between EMSs and specialist physicians. The results from our survey confirms that the majority of European countries do have organized systems for stroke emergency care in place but with significant gaps in some countries and in some important aspects of care. Contrary to our findings, a survey among delegates at an international conference of emergency care physicians in 2016 showed little difference between adherence to American Heart Association guidelines for prehospital stroke care between high- and low-income countries with neither groups performing well.^28^ A South American survey of 25 specialists from 12 countries included some aspects of prehospital care and showed that in 58% of the countries prehospital care was not properly structured and the training of professionals for stroke care was limited, especially at the primary health care level.^29^

This study has significant limitations. While the response rate was low and there are many countries where services have not been described, there is a sufficiently large sample from a range of countries to provide what is likely to be a representative picture to enable us to recognize the areas that need urgent improvement. The majority of respondents were neurologists, and we have no way of verifying whether their reporting on the status of prehospital care was accurate, particularly regarding aspects of the training of EMS staff. Future surveys should attempt to also gain information directly from the EMSs. The responses were not formally validated, but when there were multiple returns from countries, the answers to the questions were broadly compatible with each other. The survey should be treated as qualitative evidence rather than a quantitative study. It does provide the WSO taskforce on prehospital care with a clear set of objectives for its work.

In conclusion, significant deficiencies in stroke prehospital care exist globally, especially in the LMIC. The results clearly indicated that more attention and resources are needed internationally to improve prehospital stroke care to reduce the risk of death and disability. Improvements in prehospital stroke care should develop in parallel with improvements in acute hospital stroke services. Future work needs to demonstrate how stroke outcomes worldwide can be significantly improved by improving the quantity and quality of EMSs and the organization of prehospital care, so that existing evidence-based treatments such as stroke unit care, restoration of homeostasis, and revascularization can be delivered.

## Supplemental Material

sj-pdf-1-wso-10.1177_17474930231177204 – Supplemental material for Results of an international survey on the status of prehospital care Click here for additional data file. Supplemental material, sj-pdf-1-wso-10.1177_17474930231177204 for Results of an international survey on the status of prehospital care by Anthony G Rudd, Jing Zhao, Gary Ford, Rita Melifonwu, Siju V Abraham, Marc Fisher, Grethe Andersen, David Waters, Dou Li and Renyu Liu in International Journal of Stroke
