# Supplementary material for: Results of an international survey on the status of prehospital care
Source: Int J Stroke. 2023 May 30;18(9):1084–91. doi: 10.1177/17474930231177204 (PMC10614170; doi:10.1177/17474930231177204)
Supplement: sj-pdf-1-wso-10.1177_17474930231177204 – Supplemental material for Results of an international survey on the status of prehospital care [file sj-pdf-1-wso-10.1177_17474930231177204.pdf]

Supplemental Table 3. Responses by country or region

Country/Region	HIC or LMIC	Number of respondents	Access to ambulance service	Proportion of patients arriving at hospital by ambulance (%)	Payment required to use ambulance	Average ambulance response time (mins)	% patient arriving within 3 hours	% patients arriving after 24 hours	Presence of specialist stroke centres	Proportion taken directly to stroke centre (%)	Training for call handlers	Training for paramedics	Training for primary care
**AFRICA**													
Egypt	LMIC	5	Yes	10-50	2 yes, 3 no	20-45	15-28	18-60	Yes	10-40	3 of the 5 responses	None	1 yes , 4 no
Mozambique	LMIC	1	No	N/A	N/A	N/A	<5	>50	No	N/A	No	No	No
Nigeria	LMIC	7	6 no, 1 yes	<5	Yes	300-1440	0.5-20	80-90	No	N/A	No	2 yes, 5 no	No
Oman	HIC	1	Yes	60	No	60	70	30	Yes	70	Yes	Yes	Yes
Rwanda	LMIC	3	Yes	5	Yes	20-120	2-60	20-30	No	N/A	No	1 yes, 2 No	2 yes, 1 no
Senegal	LMIC	1	Yes	2-3	Yes	60-120	1	50	Yes	5	No	Yes	No
Tanzania/Zanzibar	LMIC	2	No	N/A	Yes	N/A	<5	20-50	No	N/A	No	No	No
Tunisia	LMIC	1	Yes	100	No	60	25	10	Yes	10	No	No	No
Zambia	LMIC	1	No	N/A	N/A	N/A	<5	50	No	N/A	No	No	No
**NORTH AMERICA**													
Canada	HIC	3	Yes	70-80	1 yes, 2 No	7-15	20-95	1-30	Yes	95	Yes	Yes	Yes
USA	HIC	9	Yes	40-80	6 Yes, 2 No	10-30	10-60	10-40	Yes	80-100	Yes	Yes	Yes
**ASIA**													
China	LMIC	13	Yes	20-60	1 yes, 12 no	5-40	5-30	15-90	Yes	30-90	Yes	Yes	Yes
India	LMIC	8	8 yes, 1 no	10-70	7 yes,1 no	10-120	1-60	10-90	4 No, 4 yes.	0-35	4 yes, 4 No	3 Yes, 5 No	3 Yes, 5 No
Indonesia (Bali)	LMIC	1	Yes	<5	Yes	60	5	30	No	N/A	No	No	No
Japan	HIC	2	Yes	100	No	5-10	50	5	Yes	95	Yes	No	Yes
Pakistan	LMIC	1	Yes	100	No	360	<3	97	No	N/A	No	Yes	No
Taiwan	HIR	1	Yes	N/K	No	N/K	N/K	N/K	Yes	N/K	No	No	No
South Korea	HIC	5	Yes	30-60	No	10-30	25-30	30	Yes	30-9=80	2 of 5	3 yes, 2 no	No
Sri Lanka	LMIC	1	Yes	10	No	30	20	60	Yes	N/K	Yes	Yes	Yes
**AUSTRALASIA**													
Australia	HIC	3	Yes	70-90	1 yes, 2 No	15-30 urban	30-40	10	Yes	80	Yes	Yes	No
New Zealand	HIC	1	Yes	70	Yes	15	30	30	Yes	80	Yes	Yes	No
**EUROPE**													
Armenia	LMIC	1	Yes	80	No	40	50	10	Yes	90	No	No	No
Austria	HIC	1	Yes	60-90	No	15	71	5	Yes	80-90	Yes	No	No
Croatia	HIC	3	Yes	70-75	No	20-30	25-40	10	Yes	60-90	2 Yes 1 No	No	1 Yes 2 No

enmark	HIC	1	Yes	N/K	No	<15	43	N/K	Yes	N/K	Yes	Yes	Yes
inland	HIC	1	Yes	95	Yes	20	50	20	Yes	70	Yes	Yes	Yes
eorgia	LMIC	1	Yes	53	No	51	30	2	Yes	N/K	Yes	No	No
ermany	HIC	2	Yes	80-90	No	8-10	25	25	Yes	90	Yes	Yes	Yes
reece	HIC	3	Yes	20-35	No	30-60	10-30	25-60	No	N/A	No	No	No
Italy	HIC	4	Yes	40-85	3 No, 1 yes	10-15	40-50	10-30	Yes	50-90	3 yes, 1 no	3 yes 1 no	1 yes, 3 no
Moldova	LMIC	1	Yes	50	No	30	5	50	Yes	50	No	No	No
Monaco	HIC	2	Yes	20-40	No	7-20	20	50	Yes	30	No	No	No
Poland	HIC	3	Yes	85-95	No	10-40	15-30	20-25	Yes	90-95	2 yes, 1 no	2 yes, 1 no	2 yes, 1 no
Portugal and Madeira	HIC	1	Yes	15-45	No	20-30	65	4-10	Yes	95	1 yes, 1 no	1 yes, 1 no	1 yes, 1 no
Serbia	LMIC	1	Yes	70	No	15	50	5	Yes	70	No	No	No
Spain	HIC	4	Yes	60-90	No	10-20	38-60	8-10	Yes	75-90	3 yes, 1 no	Yes	2 yes, 2 no
Sweden	HIC	1	Yes	76	No	20	33	4	No	N/A	Yes	Yes	Yes
Switzerland	HIC	1	Yes	60	No	15	70	1	Yes	80	Yes	No	Yes
Ukraine	LMIC	3	Yes	80-90	No	20-60	10-12	20-65	Yes	80-95	2 yes, 1 no	2 yes 1 no	2 yes, 1 no
United Kingdom	HIC	1	Yes	95	No	20	50	5	Yes	60	No	Yes	No
**SOUTH AMERICA**													
Argentina	LMIC	1	Yes	50	No	30	50	10	Yes	25	No	No	No
Brazil	LMIC	7	6 yes, 1 no	40-75	6 No, 1 Yes	20-360	5-60	10-50	6 yes, 1 no	20-100	2 yes, 5 no	4 yes, 3 no	4 yes 3 no
Colombia	LMIC	1	Yes	23	Yes	60	30	10	yes	20	No	No	No
Me ico	LMIC	2	Yes	5-40	No	30-40	10	50-70	No	N/A	1 yes, 1 no	1 yes, 1 no	1 yes, 1 no

Abbreviations
HIC. High Income Country
HIR High Income Region
LMIC. Low and Middle Income Country
N/K. Not known
N/A. Not applicable
